# Influence of Shading on Essential Oil Quantity and Quality of Sage (*Salvia officinalis* L.) at Different Harvest Times

**DOI:** 10.3390/plants15111711

**Published:** 2026-06-01

**Authors:** Lidija Milenković, Zoran S. Ilić, Ljiljana Stanojević, Ljubomir Šunić, Aleksandra Milenković, Nadica Tmušić, Dragana Lalević, Jelena Stanojević, Dragan Cvetković, Žarko Kevrešan

**Affiliations:** 1 Faculty of Agriculture, University of Priština in Kosovska Mitrovica, 38219 Lešak, Serbia; lidija.milenkovic@pr.ac.rs (L.M.); ljubomir.sunic@pr.ac.rs (L.Š.); nadica.tmusic@pr.ac.rs (N.T.); dragana.lalevic@pr.ac.rs (D.L.); 2 Faculty of Technology, University of Niš, Bulevar Oslobodenja 124, 16000 Leskovac, Serbia; ljiljas76@yahoo.com (L.S.); aleksandra.milenkovic@student.ni.ac.rs (A.M.); jelena_stanojevic@yahoo.com (J.S.); dragancvetkovic1977@yahoo.com (D.C.); 3 Institute of Food Technology, University of Novi Sad, Bulevar Cara Lazara 2, 21000 Novi Sad, Serbia; zarko.kevresan@fins.uns.ac.rs

**Keywords:** shade nets, *S. officinalis*, essential oil, components, antioxidant activity

## Abstract

The yield, chemical profile and antioxidant activity of sage (*Salvia officinalis* L.) essential oils (SEOs) isolated from shaded (pearl-, red- and blue-colored nets) or non-shaded plants from three different harvest-time phenological stages (May, August and September) investigated. Both main effects and their interactions were highly significant (*p* < 0.01). Blue nets produced the highest yield in the first (4.09 mL/100 g) and second (3.29 mL/100 g) harvests, significantly exceeding all other treatments within the same harvest period. In the third harvest, unshaded control plants achieved the highest yield (3.55 mL/100 g). The total number of individual SEO components varied depending on the harvest time and shading treatment (27–35). The most abundant components were thujone (cis-thujone, 24.1–36.1%; trans-thujone, 4.9–13.1%) camphor (20.0–30.2%) and 1,8-Cineole (8–11%). The content of undesirable component camphor was the lowest in all three harvests in plants covered with blue shading nets. FRAP values ranged from 0.462 mg Fe^2+^/g oil (second harvest, red net) to 1.151 mg Fe^2+^/g oil (first harvest, red net), while EC_50_ values ranged from 9.169 mg/mL (first harvest, red net) to 37.004 mg/mL (third harvest, blue net). The third-harvest blue-net sample exhibited one of the highest FRAP values (1.123 mg Fe^2+^/g oil) yet the weakest DPPH radical scavenging activity (EC_50_ = 37.004 mg/mL), reflecting different mechanisms of antioxidant action between the two assays. In conclusion, the highest yield and best quality of EO were achieved in the first harvest. Shading the plants, particularly with blue nets, contributed to an increase in EO yield, as well as improved EO quality, including higher thujone content and lower content of undesirable camphor. Covering the plants with blue nets during the first and second harvest periods enhanced essential oil yield and quality. In the third harvest, open-field conditions favored a higher yield; however, blue-net shading produced the lowest camphor content (20.3%), which may be advantageous for pharmaceutical applications. The choice of whether to maintain or remove nets during the third harvest should therefore be guided by the intended end use of the essential oil.

## 1. Introduction

Sage (*Salvia officinalis* L.) is a widely used species from the Lamiaceae family, valued in traditional medicine, culinary applications, and food technology. Its phytochemical composition is strongly influenced by genetic factors, environmental conditions, and cultivation practices [1]. Among environmental factors, light and temperature play key roles in plant growth, physiological processes, and the biosynthesis of phenolic compounds [2].

To improve plant production under changing climate conditions, shading with photoselective nets has been introduced as an effective cultivation technique [3,4,5,6]. These nets modify light intensity and spectral composition while also providing physical protection from environmental stressors. By altering light quality and increasing diffuse radiation, they can enhance physiological responses and improve light distribution within the plant canopy [3,4,7].

The use of colored shade nets has been shown to improve plant morphology, yield [8,9] and essential oil production [10]. Many medicinal and aromatic plants, including sage [11], produce higher quantities and better-quality essential oils under shaded conditions compared to full sunlight. However, plant responses vary depending on the species and net color, with blue, red, and pearl nets often producing different effects on yield and chemical composition [6]. Additionally, essential oil content and antioxidant activity depend on the growing conditions, plant origin [12], harvest time, and extraction methods [13]. Light management through photoselective shading represents a promising approach for improving productivity and quality in sage cultivation.

Three harvests per year with a last cut no later than early September at a height of 15 cm, followed by stubble shortening to about 5 cm in the spring, favor the productivity, persistence and quality of sage in mountain areas [14].

Harvest time affects the sage essential oil (EO) content. During the harvest period from June to September, the highest oil yield is achieved in July (3.24%). Sage essential oils distilled from leaves harvested in the early season (June) previously exhibited the highest antioxidant activity [15].

Since the main purpose of sage cultivation is the use of its EO in various industries, evaluating both EO yield and quality at harvest is of great importance for producers. Daily variations in the phytochemical composition of sage EO have also been observed, with the highest EO content (1.14%) recorded when harvesting between 4:00 and 6:00 p.m., while the lowest EO content (0.59%) was found when harvesting between 4:00 and 6:00 a.m. [16].

The combined effects of net color and harvest timing on sage EO yield, composition, and bioactivity have not been thoroughly investigated. Blue, red, and pearl nets were selected because they modify distinct regions of the light spectrum while maintaining the same shading intensity (40%), enabling a comparison of spectral quality effects on secondary metabolism. We hypothesized that shade-net color would differentially affect essential oil yield and composition, with these effects varying across harvest times due to seasonal changes in the light environment and plant phenological stage. The aim of this study was to evaluate the influence of pearl, blue, and red shade nets on the essential oil yield, chemical composition, and antioxidant activity of S. officinalis across three harvest periods.

## 2. Materials and Methods

### 2.1. Plant Material and Experimental Design

The experiment was carried out during 2024 in Moravac (southern Serbia; 43°30′ N, 21°42′ E; 159 m a.s.l.) using sage (*Salvia officinalis* L.) of a commercial variety obtained from seed company Seme-Semena (Belgrade, Serbia). Seeds were sown in spring 2022 in 104-cell plug trays, and seedlings were transplanted to the experimental field at the 5–6-leaf stage. As sage is a perennial species, plants were maintained in the field, and the experiment was conducted during the third year of growth (2024). The experiment was conducted using a split-plot arrangement within a randomized complete block design (RCBD) with three replications. Each experimental plot measured 2.5 m × 5 m and consisted of three rows, with plants spaced 30 cm apart within rows and 70 cm between rows, corresponding to 47,000 plants per hectare. No significant incidence of pests or diseases was observed during plant development. The aerial parts of sage were harvested at three phenological stages—at the beginning of flowering, upon full leaf development, and fruit set—during September 2024.

### 2.2. Cultivation Conditions and Environmental Monitoring

Irrigation was provided by a drip system during periods of drought (June–September). Irrigation was carried out using lateral polyethylene pipes (16 mm in diameter) with inline emitters paced at 50 cm, delivering water at a rate of 2 L h^−1^ under a pressure of 1 atm. To mitigate high temperature and excessive radiation, pearl, red and blue shade nets (Polysack, Nir Yitzhak, Negev Israel) with 40% shading intensity were installed 2 m above the canopy. Non-shaded plants (grown in the open field) were used as the control. Environmental parameters, including photosynthetically active radiation (PAR), solar irradiance, air temperature, and relative humidity, were recorded throughout the growing season. PAR above the canopy was measured using a ceptometer (SunScan SS1, Delta-T Devices, Cambridge, UK) and expressed as PAR quantum flux (µmol m^−2^ s^−1^). Measurements were taken at several time points during the day (from 6 h to 18 h) under cloudless conditions and repeated weekly throughout the growing season (June–September). Solar irradiation was recorded using a portable solarimeter (SL 100, KIMO, Montpon, France; 1–1300 W m^−2^). Air temperature, relative humidity and light (PAR) under each shading net and in the open field were monitored using data loggers (Spectrum Technologies Inc. WatchDog model 2475 Plant Growth Station, Chicago, IL, USA) positioned at canopy height, recording at 60 min intervals throughout the experimental period.

### 2.3. Harvest and Yield Assessment

Sage plants were harvested at three distinct phenological stages during the 2024 growing season. The first harvest was carried out on 28 May at the stage of full flowering, when more than 90% of plants had open inflorescences across all treatments. The second harvest took place on 20 August, during the period of full vegetative regrowth following the first cut, characterized by fully developed leaves without secondary flowering. The third harvest was performed on 20 September at the stage of fruit set. At each harvest, the aerial parts of the plants were cut at a height of 10–15 cm above the soil surface. The phenological stage at each harvest directly influences essential oil biosynthesis, as glandular trichome density and terpene accumulation are known to peak during flowering and decline during vegetative and reproductive stages. Yield is expressed on an area basis (t ha^−1^).

Pruning was performed manually by cutting the plants to 10–15 cm above the soil surface to maintain plant architecture. The fresh biomass was immediately measured, then placed in a shaded and well-ventilated area for drying. After a drying period of three weeks, the plant material was reweighed to determine the fresh-to-dry mass ratio of *Salvia officinalis* L., which is an important parameter of this study. The same procedure was applied for all three sampling dates.

### 2.4. Environmental Data

Environmental monitoring showed that all three shade nets reduced PAR and solar irradiance compared to the open-field control throughout the growing season (Table 1). The blue net consistently produced the lowest PAR values (770.1–1018.3 µmol m^−2^ s^−1^) and the lowest solar irradiance (331.7–563.8 W/m^2^) across all months, while the red net transmitted more radiation than the pearl net. Under open field conditions, PAR peaked in July (2025.8 µmol m^−2^ s^−1^) and declined toward August (1867.4 µmol m^−2^ s^−1^). All nets reduced PAR by approximately 42–50% relative to the control, with blue nets showing the greatest reduction.

### 2.5. Essential Oil Isolation and Analysis

Disintegrated and homogenized air-dried sage leaves (80–100 g per extraction, depending on sample availability) were subjected to hydrodistillation using a Clevenger-type apparatus with a plant material-to-water ratio of 1:10 (*m*/*v*) for 120 min (Ph. Jug. V, 2000). The volume of separated oil was read from the graduated Clevenger tube at 15 min intervals during distillation to monitor the kinetics of oil recovery. All extractions were performed in triplicate. After distillation, the essential oil was separated from the collecting tube, dried over anhydrous sodium sulfate, and stored in sealed dark glass vials at +4 °C until analysis. The essential oil yield was calculated as EO yield (mL/100 g) = (volume of oil obtained/mass of dry plant material) × 100 and expressed in mL per 100 g of air-dried plant material.

Qualitative analysis of the essential oils was performed on an Agilent Technologies 7890B gas chromatograph equipped with a nonpolar HP-5MS capillary column (5% diphenyl and 95% dimethyl-polysiloxane, 30 m × 0.25 mm i.d., 0.25 μm film thickness; Agilent Technologies, Santa Clara, CA, USA) coupled with an Agilent 5977A inert selective mass detector. Samples were dissolved in diethyl ether, and 1 μL was injected through a split/splitless inlet at 220 °C in 40:1 split mode. Helium was used as the carrier gas at a constant flow rate of 1 mL/min. The oven temperature was programmed from 60 °C to 246 °C at a rate of 3 °C/min. The MSD transfer line, ion source, and quadrupole mass analyzer temperatures were set at 300 °C, 230 °C, and 150 °C, respectively. Electron ionization was performed at 70 eV, with a mass scan range of *m*/*z* 41–415.

Semi-quantitative analysis was carried out by GC/FID under identical chromatographic conditions on the same instrument. The carrier gas (He), make-up gas (N_2_), fuel gas (H_2_), and oxidizing gas (air) flow rates were 1, 25, 30, and 400 mL/min, respectively. The FID temperature was set at 300 °C. The content of individual components was determined by the area normalization method of the GC/FID signal without correction factors.

Data processing was performed using MSD ChemStation (version F.01.00.1903), AMDIS (version 2.70), and NIST MS Search (version 2.0g) software. Retention indices were experimentally determined using homologous series of n-alkanes (C_8_–C_20_ and C_21_–C_40_) as standards. Compound identification was based on comparison of experimental retention indices (RI_exp) with literature values (Adams, 2007) [17]; mass spectral matching against Wiley 6, NIST 2011, and RTLPEST3 libraries; and, where available, co-injection with authentic standards [17].

### 2.6. Determination of Antioxidant Activity

#### 2.6.1. DPPH Radical Scavenging Assay

The ability of sage essential oils to scavenge free DPPH radicals was determined according to Stanojević et al. [18]. Essential oils were dissolved in ethanol, and a series of concentrations was prepared (0.781–25 mg/mL). Ethanolic DPPH radical solution (1 mL, 3 × 10^−4^ mol/dm^3^) was added to 2.5 mL of each essential oil solution. After incubation for 20 min at room temperature, absorbance was measured at 517 nm using a Perkin Elmer Lambda 25 spectrophotometer (PerkinElmer, Inc., Waltham, MA, USA). Free radical scavenging activity (SC%) was calculated as SC (%) = (1 − (A_S − A_B)/A_C) × 100, where A_S is the absorbance of the sample (essential oil solution treated with DPPH), A_B is the absorbance of the blank (essential oil solution without DPPH), and A_C is the absorbance of the control (DPPH solution in ethanol). The EC50 value, defined as the essential oil concentration required to neutralize 50% of the initial DPPH radical concentration, was determined by linear regression analysis of the SC% versus concentration curve.

#### 2.6.2. FRAP Assay

The ferric reducing ability was determined according to Benzie and Strain [19] with modifications described by Stanojević et al. [20]. Ethanolic solutions of sage essential oil (0.1 cm^3^) were mixed with 3 cm^3^ of freshly prepared FRAP reagent and incubated at 37 °C in a water bath for 30 min. Absorbance was measured at 593 nm against a blank (3 cm^3^ FRAP reagent + 0.1 cm^3^ of 96% *v*/*v* ethanol). The concentration of Fe^2+^ equivalents was determined from a FeSO_4_·7H_2_O calibration curve (A_593_ = 0.03521 + 0.5949 × c(FeSO_4_·7H_2_O), R^2^ = 0.993) [11]. Results are expressed as mg Fe^2+^ equivalents per gram of essential oil (mg Fe^2+^/g EO).

### 2.7. Statistical Analysis

All measurements were performed in triplicate. Data were analyzed using two-way analysis of variance (ANOVA), with harvest time and shade net treatment as fixed factors, including their interaction. Mean comparisons were conducted using Duncan’s multiple range test at *p* < 0.05. Principal component analysis (PCA) was applied to explore the multivariate relationships among essential oil yield, antioxidant activity parameters (FRAP and EC_50_), and essential oil composition. Prior to PCA, trace amounts (<0.05%) were replaced with 0.025, non-detected compounds were set to zero, variables with negligible variation across samples were excluded, and the remaining data were autoscaled (mean-centered and divided by standard deviation) to give equal weight to variables measured on different scales. Pearson’s correlation coefficients were calculated among essential oil yield, antioxidant activity parameters, and major essential oil components to evaluate pairwise relationships. The statistical significance of correlations was assessed at *p* < 0.05 and *p* < 0.01 levels. Hierarchical cluster analysis (HCA) was performed on the autoscaled data using Euclidean distance and Ward’s linkage method to assess similarity and grouping patterns among samples. Statistical analyses including PCA were performed using STATISTICA 14 TIBCO Software, Inc., Palo Alto, CA, USA (2020). Data Science Workbench, version 14.

## 3. Results

### 3.1. Leaf Morphology and Sage Yield

Leaf morphology was significantly affected by shade treatment. Plants grown under red and blue shade nets had the largest leaves, while the leaves of plants grown under full sunlight were the smallest. Shading had a significant effect on plant height (*p* < 0.05), with plants grown under red and blue nets being taller than grown under pearl nets and those grown under full sunlight being shortest. Irradiance had a clear effect on fresh biomass. The highest fresh biomass was obtained in plants grown under red and blue nets, with dry biomass exhibiting a similar response (Table 2). The mean values of the fresh/dry ratio during the four-month experiment are shown in Table 2. The fresh/dry ratio was highest under blue nets, followed by red and pearl nets, and lowest in unshaded plants.

The dry herb yield obtained in late August was approximately 80% higher than that from the harvest carried out in late May (Table 1). Similarly, the dry leaf yield was approximately 70% higher in August than in May. Studies have shown that the yield of essential oil achieved from leaves harvested in May is significantly lower than that obtained in August. The lowest fresh and dry sage biomass yields were recorded at the third harvest in September, at the end of the growing season, before the onset of winter dormancy.

### 3.2. Essential Oil Yield

Essential oil contents may depend on climatic conditions such as soil, temperature, and light intensity, or they may be genetically determined. Light, in particular, has a significant effect on both the content and composition of sage EO. The use of colored shade nets, which modify light quantity and quality, can influence EO yield and composition depending on the harvest time and phenological stage of the plant. Thus, seasonal variability in sage EO composition was clearly demonstrated.

Essential oil yield varied significantly among all 12 harvests× shade net combinations, ranging from 1.63 mL/100 g of plant material (harvest III, red net) to 4.09 mL/100 g (harvest I, blue net) (Table 3).

Both main effects and their interaction were highly significant (*p* < 0.01). Blue nets produced the highest yield in the first (4.09 mL/100 g) and second (3.29 mL/100 g) harvests, significantly exceeding all other treatments within the same harvest period. In the third harvest, however, the pattern reversed: unshaded control plants achieved the highest yield (3.55 mL/100 g), while the blue-net yield dropped to 1.87 mL/100 g, which is comparable to the red-net treatment. Red nets and pearl nets generally produced yields similar to the control in the first harvest, but yields declined across successive harvests (Table 3).

The significant interaction effect indicates that the influence of net color on oil accumulation is strongly dependent on harvest timing, likely reflecting differences in plant phenological stage, light quality requirements during oil biosynthesis, and seasonal shifts in temperature and radiation intensity [21]. The superior performance of blue nets in earlier harvests may be related to enhanced diffuse blue-spectrum radiation promoting glandular trichome development and monoterpene biosynthesis during the active flowering stage, while the decline in later harvests suggests that post-flowering sage plants may benefit from higher total irradiance available under open-field conditions.

### 3.3. Essential Oil Composition

The composition of the essential oils of sage depended on the harvest time and the light conditions. Among total constituents in the first harvest (27–33), the key constituents in SEO were thujone (cis-thujone, 27.2–31.6%; trans-thujone, 5.5–8.0%), camphor (22.8–29.9%) and 1,8-cineole (9–11%).

The highest number of total identified components in SEO from the first harvest was recorded in plants covered with blue nets (33), while the lowest number (27) was observed under pearl nets. The most abundant component, was thujone ranging from 39.5% (*cis*-thujone, 31.6%; *trans*-thujone, 7.9%) in plants cover by blue nets to 32.8% (*cis*-tujone 27.3%; 5.5%, *trans*-thujone) in plants covered with red nets.

The second most abundant component was camphor, a component known to reduce essential oil quality, accounting for 22.8% in plants grown under blue nets, with the highest content (29.9%) recorded under red nets and in control (non-shaded) plants (29.1%). 1,8-Cineole was the third most abundant component, ranging from 9% in non-shaded plants to 11% in plants shaded with pearl nets (Table 4).

Oxygen-containing monoterpenes were the most dominant group in SEO, ranging from 77.1% under blue nets to 81.6% under pearl shading nets. Monoterpene hydrocarbons (9.9–12.2%), oxygen-containing sesquiterpenes (2.3–4.6%) and sesquiterpene hydrocarbons (3.2–6.0%), as well as other components like diterpenes (0.7–1.9%), were present to a much lesser extent (Table 4).

During the initial flowering stage, sage plants grown in the open field contained 33 components in total. In the first harvest, several components were absent from the SEO of plants covered with pearl nets: camphene, (*Z*)-β-ocimene, *cis*-sabinene hydrate, iso-3-thujanol, aromadendrene and myrtenol. In plants covered with red nets during the same harvest, iso-3-thujanol and (*Z*)-β-ocimene were absent.

Among total constituents in the second harvest (32–34), the key constituents in SEO were thujone (cis-thujone, 24.1–35.0%; trans-thujone, 4.9–13.1%), camphor (24.7–30.2%) and 1,8-cineole (8.1–10.8%) (Table 5).

The highest number of total identified components in SEO from the second harvest was recorded in non-shaded plants (35), while the lowest number (33) was observed in plants covered by blue nets. The most abundant component was thujone ranging from 43.5% (*cis*-thujone, 35.0%; *trans*-thujone, 8.5%) in plants covered by red nets to 32.3% (*cis*-tujone, 27.4%; *trans*-thujone, 4.9%) in non-shaded control plants.

The second most abundant component was camphor, a component known to reduce essential oil quality, accounting for 24.7% in plants grown under blue nets, with the highest content (30.2%) recorded in unshaded control plants (30.2%). 1,8-cineole was the third most abundant component, ranging from 8.1% in plants shaded with red nets to 10.8% in non-shaded plants (Table 5).

Oxygen-containing monoterpenes were the most dominant group in SEO, ranging from 76.0% under blue nets to 81.3% under red nets. Monoterpene hydrocarbons (10.7–12.6%), oxygen-containing sesquiterpenes (2.8–5.5%) and sesquiterpene hydrocarbons (3.2–4.7%), as well as other components like diterpenes (1.3–2.9%), were present to a much lesser extent (Table 5).

Among the total constituents in the third harvest (33–35), the key components were thujone (cis-thujone, 28.0–36.1%; trans-thujone, 6.6–9.6%), camphor (20.0–23.4%) and 1,8-Cineole (8.0–10.6), (Table 6).

The highest number of total identified components in SEO from the third harvest was recorded in plants covered by blue and pearl nets (34), while the lowest number (32) was observed under red nets. The most abundant component was thujone, ranging from 42.7% (*cis*-thujone, 36.1%; *trans*-thujone, 6.6%) in plants covered by red nets to 37.6% (*cis*-thujone, 28.0%; *trans*-thujone, 9.6%,) in non-shaded control plants.

The second most abundant component was camphor, a component known to reduce essential oil quality, accounting for 20.0% in plants grown under blue nets, with the highest content (23.4%) recorded in control (non-shaded) plants (30.2%). 1,8-Cineole was the third most abundant component, ranging from 8.0% in plants shaded with pearl nets to 10.6% in plants covered by blue nets (Table 6).

Oxygen-containing monoterpenes were the most dominant group in SEO from the third harvest, ranging from 74.2% in non-shaded plants to 78.9% under red shading nets. Monoterpene hydrocarbons (10.8–14.3%), sesquiterpene hydrocarbons (3.4–7.6%), and oxygen-containing sesquiterpenes (3.5–4.8%), as well as other components like diterpenes (1.0–2.3%), were present to a much lesser extent (Table 6).

The contents of seven major essential oil compounds were statistically evaluated across all harvest × net treatment combinations (Table 7). Both main effects and their interactions were highly significant (*p* < 0.01) for all compounds. cis-Thujone ranged from 24.0% (second harvest, pearl) to 36.4% (third harvest, red), with the highest values recorded under red and blue nets in the third harvest. Camphor ranged from 20.3% (third harvest, blue) to 30.2% (second harvest, control), with blue nets consistently producing the lowest camphor contents across all three harvests. 1,8-Cineole ranged from 8.1% (third harvest, pearl) to 10.8% (second harvest, control), showing less pronounced treatment effects than thujone and camphor. The full ANOVA results are presented in Supplementary Table S1.

### 3.4. Antioxidant Activity

The FRAP values of the analyzed essential oil samples ranged from 0.462 ± 0.002 to 1.151 ± 0.005 mg EFe^2+^/g oil, indicating differences in reducing capacity among the samples. The highest FRAP value was recorded in harvest I under red nets, followed by harvest III under blue nets, suggesting a higher antioxidant potential in these plant samples. In contrast, the lowest values were observed in harvest II under red nets and harvest II under pearl nets.

The EC50 values after 20 min ranged from 9.169 to 37.004 mg/mL, indicating differences in antioxidant activity among the essential oil samples. The lowest EC50 value, corresponding to the highest activity, was observed in the first harvest under red nets, followed by the second harvest under blue nets. In contrast, in the third harvest, plants under blue nets and red nets showed the highest EC50 values, indicating lower antioxidant activity. Overall, no consistent pattern was observed between net shading and antioxidant activity.

The antioxidant activity of sage essential oils, as assessed by both FRAP and DPPH assays, was significantly affected by harvest time, shade net treatment, and their interaction (*p* < 0.01; Table 8). However, the two assays revealed contrasting patterns, reflecting different aspects of antioxidant capacity.

The FRAP assay, which measures ferric-reducing power, showed values ranging from 0.462 mg Fe^2+^/g oil (harvest II, red net) to 1.151 mg Fe^2+^/g oil (harvest I, red net). The first-harvest red-net sample had the highest reducing capacity, significantly exceeding all other treatments, followed by the third-harvest blue net sample (1.123 mg Fe^2+^/g oil) and pearl-net sample (0.951 mg Fe^2+^/g oil). In contrast, second-harvest samples generally exhibited the lowest FRAP values, regardless of net color, with plants under red nets and pearl nets, along with the control, forming a statistically homogeneous group (0.462–0.507 mg Fe^2+^/g oil). This suggests that the reducing potential of sage essential oil is more strongly influenced by harvest timing than by shade treatment alone.

The DPPH radical scavenging activity, expressed as EC50, ranged from 9.169 mg/mL (harvest I, red net) to 37.004 mg/mL (harvest III, blue net), with lower values indicating stronger activity. The first-harvest red-net sample showed, by far, the highest radical scavenging capacity, consistent with its superior FRAP value. In the second harvest, blue nets produced the most active oil (EC50 = 13.391 mg/mL)—significantly better than all other second-harvest treatments. Notably, the third harvest showed the weakest DPPH activity across all net treatments, with blue and red nets recording the highest EC50 values (37.004 and 36.677 mg/mL, respectively), in stark contrast to their strong FRAP performance in the same harvest.

The divergent trends between the two assays in the third harvest suggest that the essential oil composition at this developmental stage favors electron transfer-based reducing mechanisms (captured by FRAP) rather than hydrogen atom transfer-based radical scavenging (captured by DPPH). This is consistent with the higher proportion of oxygenated sesquiterpenes such as viridiflorol and humuleneepoxide II detected in the third harvest (Table 3, Table 4 and Table 5), which are known to exhibit reducing activity but variable radical scavenging efficiency. The significant interaction effect confirms that no single net treatment consistently optimizes antioxidant activity across all harvests and assay types, underscoring the importance of matching harvest timing to the desired bioactivity profile.

**Figure 1:**
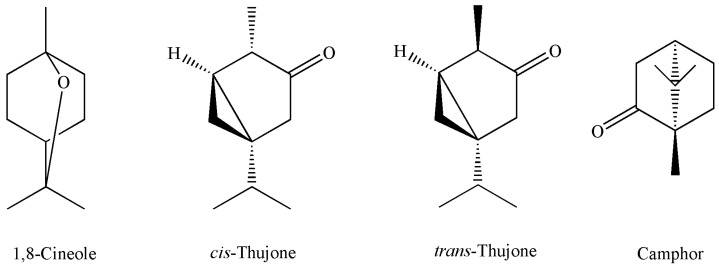
Chemical structures of the most abundant components in sage essential oil.

**Figure 2:**
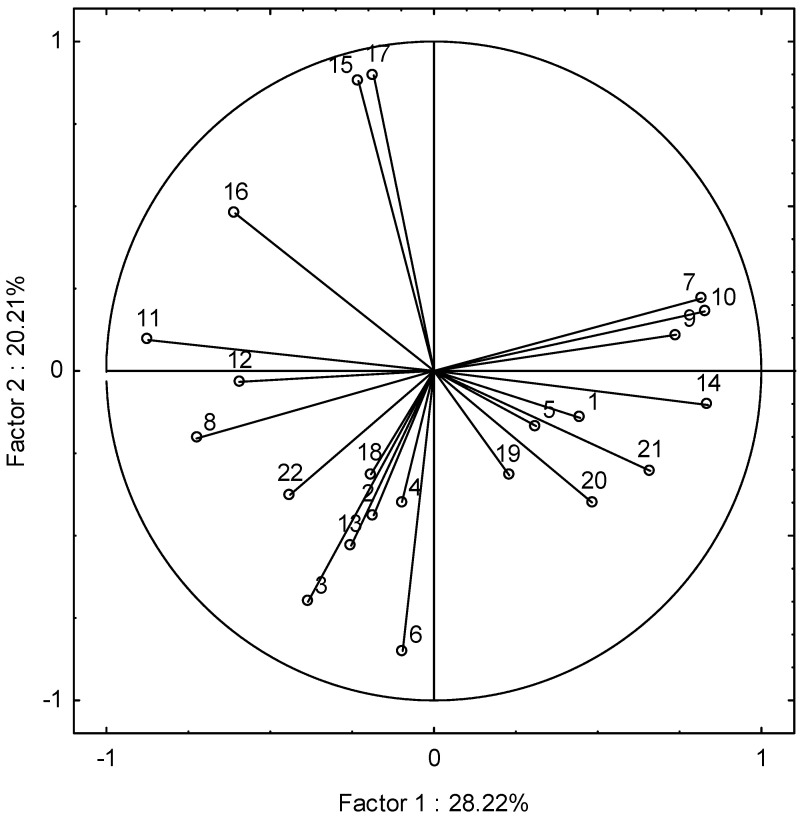
Principal component analysis (PCA) loading plot of variables projected on the PC1 × PC2 factor plane. Variables: 1—essential oil yield (mL/100 g p.m.); 2—FRAP value (mg Fe^2+^/g oil); 3—EC_50_ (mg/mL); 4—α-pinene; 5—camphene; 6—β-pinene; 7—1-octen-3-ol; 8—myrcene; 9—α-terpinene; 10—*o*-cymene; 11—limonene; 12—1,8-cineole; 13—*cis*-thujone; 14—*trans*-thujone; 15—camphor; 16—borneol; 17—bornyl acetate; 18—(*E*)-caryophyllene; 19—α-humulene; 20—viridiflorol; 21—humulene epoxide II; 22—manool.

Principal component analysis was performed on 22 variables (essential oil yield, FRAP, EC50, and 19 individual essential oil compounds) across the 12 harvests × net treatment combinations. The first two principal components accounted for 48.4% of the total variance (PC1: 28.2%; PC2: 20.2%) and were used to visualize the relationships among variables and to assess the separation of samples (Figure 1 and Figure 2).

The variable loading plot (Figure 2) revealed that PC1 was positively associated with trans-thujone, o-cymene, 1-octen-3-ol, α-terpinene, humulene epoxide II, and viridiflorol on the right side, while limonene, myrcene, 1,8-cineole, and manool were positioned on the left, indicating a negative contribution. This axis primarily reflects a contrast between volatile monoterpene alcohols and hydrocarbons. PC2 was positively loaded by bornyl acetate, camphor, and borneol in the upper region, while EC50, β-pinene, cis-thujone, and FRAP values contributed negatively in the lower region. PC2 appears to be associated with variation in the oxygenated monoterpene fraction and antioxidant properties, although this interpretation should be considered with caution, given that the first two components explain 48.4% of the total variance.

**Figure 3:**
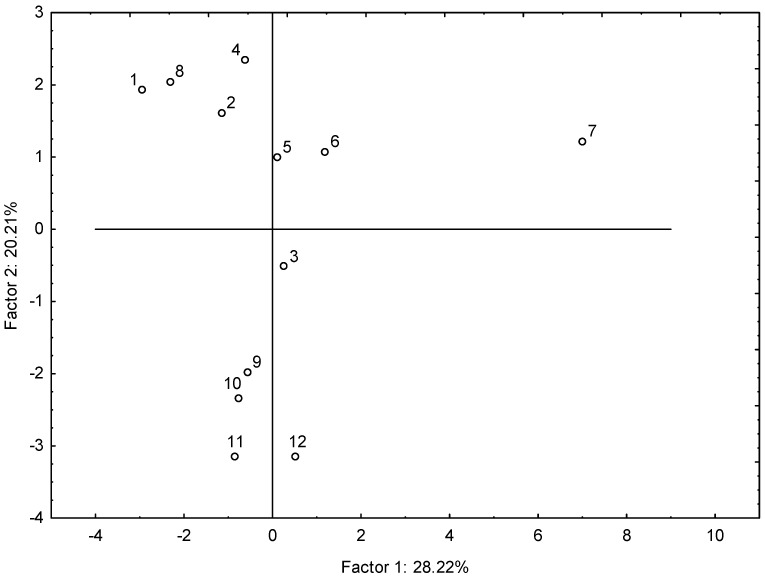
Principal component analysis (PCA) score plot of sage (*Salvia officinalis* L.) essential oil samples projected on the PC1 × PC2 factor plane. Samples: 1—H1 Pearl; 2—H1 Red; 3—H1 Blue; 4—H1 Control; 5—H2 Pearl; 6—H2 Red; 7—H2 Blue; 8—H2 Control; 9—H3 Pearl; 10—H3 Red; 11—H3 Blue; 12—H3 Control. H1, H2, and H3 denote first, second, and third harvest, respectively.

The case score plot (Figure 3) showed a general separation of samples along PC2, with most first- and second-harvest samples (points 1, 2, 4, 5, 6, 8) positioned in the upper half of the plot (positive PC2) and all third-harvest samples (points 9–12) grouped in the lower half (negative PC2). A notable exception was H1 Blue (point 3), which, despite being a first-harvest sample, was positioned in the negative PC2 region. This separation from the other first-harvest treatments reflects the distinct compositional profile of H1 Blue, which had the lowest camphor content (22.8%) and highest combined thujone content (39.5%) among all first-harvest samples, shifting it toward the third-harvest cluster, where lower camphor and higher sesquiterpene proportions were characteristic. H2 Blue (point 7) was positioned far to the right on PC1, clearly separated from all other samples. This position reflects its combination of high essential oil yield (3.29 mL/100 g) and elevated o-cymene, trans-thujone, and α-terpinene, together with reduced limonene, 1,8-cineole, and camphor, which are the variables with the strongest contributions to PC1, as shown in the loading plot. In the first harvest, H1 Pearl (point 1) and H1 Control (point 4) occupied the upper-left quadrant, associated with higher camphor and bornyl acetate contents, while H1 Red (point 2) was positioned slightly to the right, reflecting its higher FRAP value and stronger antioxidant capacity. Among third-harvest samples, H3 Control (point 12) shifted notably to the right compared to the other third-harvest treatments, consistent with its highest essential oil yield in that harvest period.

Overall, the PCA was consistent with the significant interaction between harvest time and shade-net treatment observed in the ANOVA. The separation of samples along PC2 indicates that harvest timing contributed to differentiation of the overall phytochemical profile, while net treatment effects were visible within harvest periods. However, since the first two principal components account for 48.4% of total variance, the remaining variability is distributed across additional components, and the biological interpretations drawn from the PC1 × PC2 biplot should be considered indicative rather than conclusive.

Pearson’s correlation analysis was performed to evaluate the pairwise relationships among essential oil yield, antioxidant activity, and major essential oil components (Table 9). Essential oil yield showed a significant positive correlation with α-humulene (r = 0.60, *p* < 0.05), while no significant relationship was found between yield and either antioxidant parameter. EC_50_ was significantly positively correlated with cis-thujone (r = 0.61, *p* < 0.05) and negatively correlated with camphor (r = −0.62, *p* < 0.05), indicating that samples with higher cis-thujone and lower camphor contents exhibited weaker radical scavenging activity. Camphor and cis-thujone showed a significant inverse relationship (r = −0.64, *p* < 0.05), confirming their opposing biosynthetic trends across treatments. Among sesquiterpenes, viridiflorol and humulene epoxide II were very strongly correlated (r = 0.95, *p* < 0.01), suggesting a shared biosynthetic origin. Trans-thujone was significantly correlated with humulene epoxide II (r = 0.62, *p* < 0.05) and negatively correlated with 1,8-cineole (r = −0.59, *p* < 0.05). These correlations support the compositional groupings observed in the PCA and provide additional evidence for harvest-dependent shifts in terpene metabolism.

**Figure 4:**
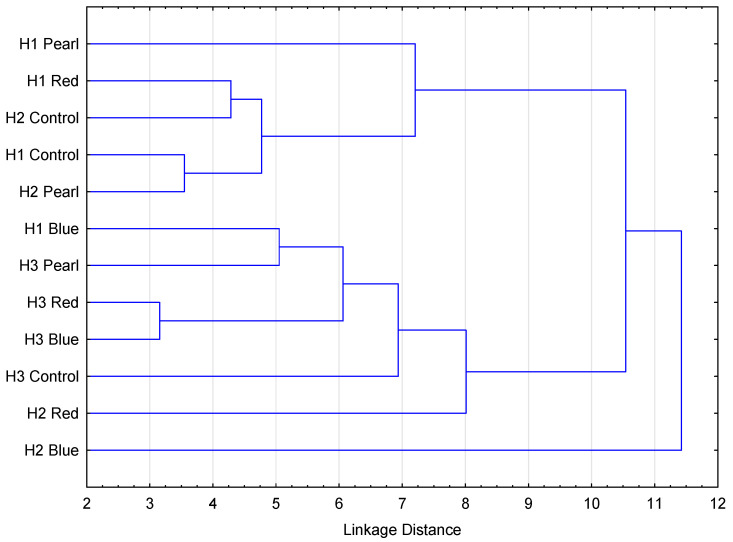
Dendrogram of hierarchical cluster analysis (HCA) of 12 sage (*Salvia officinalis* L.) essential oil samples using Ward’s method and Euclidean distances on auto scaled data. H1, H2, and H3—first, second, and third harvest, respectively.

Hierarchical cluster analysis (HCA) using Ward’s method and Euclidean distances revealed three main clusters among the 12 samples (Figure 4). The first cluster grouped H1 Pearl, H1 Red, H1 Control, H2 Control, and H2 Pearl, representing samples characterized by higher camphor content and moderate essential oil yields. The second cluster comprised all four third-harvest samples, together with H1 Blue, reflecting their shared compositional features, including lower camphor and higher sesquiterpene proportions. The separation of H1 Blue from the other first-harvest treatments confirms its distinct phytochemical profile observed in the PCA. The third cluster contained H2 Red and H2 Blue, with H2 Blue showing the greatest dissimilarity from all other samples (linkage distance of ~12), consistent with its outlier position on PC1. The HCA groupings are in general agreement with the PCA score plot, confirming that harvest time is the primary factor driving sample differentiation, while shade-net effects are most pronounced under the blue-net treatment.

## 4. Discussion

Environmental factors such as light, humidity, temperature, altitude, and soil conditions can cause biochemical, physiological, and phenotypic changes that influence the content and composition of secondary metabolites, including essential oils (EOs). Among these factors, light is considered one of the most important, as it strongly affects EO metabolism and composition in medicinal plants.

The environmental data (Table 1) provide context for interpreting the observed differences in essential oil yield and composition across harvests. The highest PAR levels were recorded in June and July, coinciding with the first harvest period, when essential oil yield was at its maximum, particularly under blue nets. Despite receiving the lowest absolute PAR among the shaded treatments, blue-net plants produced the highest essential oil yield in the first and second harvests, suggesting that the spectral quality of transmitted light was the primary driver of enhanced terpene accumulation rather than total photon flux. The progressive decline in PAR from July to August, combined with the additional shading effect, may explain the reduced essential oil yields observed in the second and third harvests under all net treatments. In the third harvest, when ambient PAR was already declining due to shorter day length and lower solar angle, the additional shading became counterproductive, resulting in open-field plants outperforming all shaded treatments in essential oil yield.

High solar radiation and elevated summer temperatures can reduce both plant yield and quality. Photo selective shade nets containing reflective and light-dispersive additives convert direct sunlight into diffuse radiation, improving light penetration within the plant canopy. Their radiometric properties depend mainly on net color and porosity [22]. By modifying the light quality and spectrum, colored shade nets influence photoreceptors such as phytochromes and cryptochromes, which regulate photosynthesis, antioxidant activity, pigmentation, and the production of secondary metabolites, including phenols, flavonoids, and essential oils. These changes often enhance the phytochemical composition and medicinal quality of aromatic and medicinal plants [23].

UV radiation also plays an important role in the accumulation of secondary metabolites, particularly phenolic compounds and flavonoids, which contribute to plant color, taste, and health-promoting properties, although studies on UVB: PAR and UVA:PAR ratios under horticultural conditions remain limited. The content of sage EO depended on both light conditions and harvest time. The highest yield of sage essential oil (SEO) was achieved in the first harvest, at the pre-flowering stage at the end of May, compared to the other two harvests. The greatest SEO yield was obtained from plants covered with blue shade nets (4.09%). A similar trend was observed in the second harvest, where the highest yield was also recorded in plants grown under blue nets (3.29%). At the same time, the lowest yield was found in the control plants without shading (2.02% in the first harvest and 1.75% in the second harvest).

In the third harvest, the situation was completely reversed, with the highest yield recorded in plants grown without colored shade nets (3.55%) compared to shaded plants. This could be explained by shorter day length and reduced light intensity during that period, suggesting that shading no longer had a positive effect and may have even reduced the yield. Therefore, it may be recommended to remove shade nets from plants during this stage of cultivation.

The use of colored shade nets during the growth of different medicinal plants provides spectral changes, resulting in a higher content of EOs in sage [24], sweet basil [25], mint, thyme, oregano, marjoram, and lemon balm [3,6].

The yield of sage essential oil (SEO) reported in various literature sources from different locations is considerably lower than the SEO yield recorded in Serbia and in our results. For example, the essential oil yield in Iran was lower at all phenological stages. In results from Iran, the highest oil yield (1.7%) was obtained at the stage before blooming, and the lowest oil percentage (0.36%) was obtained at the stage of fruit set [26]. Under the climatic conditions of Spain, the average sage oil yield (*v*/*w*) was approximately 1.0%. It was higher during the initial stage and full flowering (1.1–1.5%) and lower after flowering and in the vegetative stand period (less than 1.0%) [27]. In research from Lithuania, Baranauskiene et al. observed that total essential oil (EO) content increased from May 23 to June 20 and was 0.1–0.2 in fresh and 0.4–1.0 cm^3^ 100 g^−1^ in dried herb [28].

ISO 9909 [29] for medicinal uses regulates the amounts of several constituents in sage essential oils: cis-thujone (18.0–43.0%), camphor (4.5–24.5%), 1,8 cineole (5.5–13.0%), trans-thujone (3.0–8.5%), alpha-humulene (>12.0%), alpha-pinene (1.0–6.5%), camphene (1.5–7.0%), limonene (0.5–3.0%), bornyl acetate (>2.5%) and linalool + linalyl acetate (>1%).

In agreement with our results, the main constituents of SEO collected at all harvest times and from different shading treatments were monoterpenes (trans–thujone and cis-thujone, camphor, 1,8–cineole, etc.), sesquiterpenes (caryophyllene oxide and viridiflorol), and other compounds including alkaloids and phenolics (coumarins and flavonoids) [12,30].

In our explorations, the percentage of each major constituent in SEO was found to vary during all phenological periods. Alpha-thujone, beta-thujone and borneol were observed in higher percentages during initial flowering in the first harvest period, while 1,8-cineole and camphor, as undesirable components, showed lower values in the third harvest during the vegetative stand period. In the same period, alpha-humulene was observed in higher percentages.

The content of α-thujone in EO from sage plants grown in Lithuania varied within the range of 15.2–39.7%; that of β-thujone varied within the range of 5.3–7.9%. Other important components were 1,8-cineole, camphor, borneol, α-humulene, viridiflorol and manool [28].

The main components of sage essential oil from Poland were found to be 1,8-cineole (16.08–18.04%), α-thujone (10.40–21.51%) and camphor (5.24–18.08%). Essential oil from leaves harvested during the first half of May contained more than twice as much α-thujone (21.51%) as those harvested in August (10.40%) [31].

Most of the components identified in sage essential oil from Syria belong to the category of oxygenated monoterpenes (1,8-cineole, α-thujone, camphor, β-thujone, borneol and bornyl acetals) [32]. Tayoub et al. [32] found that essential oil of *Salvia officinalis* L. from Syria contained 57.3% monoterpenes and 41.7% sesquiterpenes. The study showed higher contents of monoterpenes in oil obtained from leaves during both the May (64.35%) and August (76.48%) harvests.

The main oil constituent in sage from Spain was also found to be alpha thujone (40.1–46.5%). Other identified compounds included beta pinene (2.6–4.5%), cineole (3.5–8.7%), beta thujone (4.1–5.6%), camphor (4.1–8.0%), borneol (1.3–3.7%), alpha humulene (3.8–7.3%), viridiflorol (3.4–12.6%) and manool (0.1–4.5%). The highest yield of oil was obtained in the period of full flowering, and the highest concentration of alpha thujone was observed in the period of initial flowering [27].

Direct comparisons of essential oil yield and composition data across studies should be interpreted with caution, as these parameters are strongly influenced by genotype, plant age, soil type, climatic conditions, altitude, extraction method, and phenological stage at harvest [10]. The relatively high essential oil yields recorded in the present study (up to 4.09 mL/100 g dry plant material) compared to values typically reported in the literature (0.8–2.5 mL/100 g) may be attributable to several factors specific to our experimental conditions: the use of two-year-old established plants with well-developed root systems; the warm continental climate of southern Serbia, with high summer temperatures and solar radiation favoring terpene accumulation; harvesting at optimal phenological stages; and the 120 min hydrodistillation duration, which ensures near-complete oil recovery. Additionally, differences in whether yields are reported on a fresh- or dry-weight basis can account for substantial discrepancies between studies. Therefore, while comparisons with published data from other regions provide useful reference points, the observed differences should not be attributed solely to shade-net effects without considering these confounding factors.

The compositional shifts observed across the three harvest periods can be interpreted in relation to terpene biosynthetic pathways and seasonal environmental changes. The higher proportion of cis-thujone and trans-thujone during the first harvest coincides with the flowering stage, when monoterpene synthase activity in glandular trichomes is at its peak due to active metabolic demand for pollinator attraction and herbivore defense. The progressive increase in camphor content from the first to the second harvest under most treatments likely reflects the enzymatic conversion of borneol to camphor by borneol dehydrogenase, which accumulates during post-flowering maturation. The decline in 1,8-cineole from the second to the third harvest may be associated with reduced photosynthetic activity and lower precursor availability (geranyl diphosphate) as day length decreases toward autumn. The increase in sesquiterpene hydrocarbons (α-humulene and (E)-caryophyllene) and oxygenated sesquiterpenes (viridiflorol and humulene epoxide II) observed in the third harvest is consistent with a metabolic shift from the MEP pathway (plastidial, producing monoterpenes) toward the MVA pathway (cytosolic, producing sesquiterpenes), which is known to occur during late vegetative and senescent stages. The influence of shade nets on these trends—particularly the consistent reduction in camphor under blue nets—suggests that blue-enriched light may modulate the activity of specific oxidoreductases involved in the camphor biosynthetic branch, although this mechanism requires further investigation at the molecular level.

In our research, oxygen-containing monoterpenes were found to be the most dominant group in SEO from the first harvest, ranging from 77.1% under blue nets to 81.6% under pearl shading nets: monoterpene hydrocarbons (9.9–12.2%), oxygen-containing sesquiterpenes (2.3–4.6%) and sesquiterpene hydrocarbons (3.2–6.0%).

The contents of oxygen-containing monoterpenes from the third harvest were lower than in the first harvest. Monoterpene hydrocarbon contents in the third harvest were higher than in the first harvest, while sesquiterpene hydrocarbons and oxygen-containing sesquiterpenes were present at same level in the first and third harvests.

FRAP values ranged from 0.462 mg Fe^2+^/g oil (second harvest, red net) to 1.151 mg Fe^2+^/g oil (first harvest, red net). The highest reducing capacity was recorded in the first harvest under red nets, followed by the third harvest under blue and pearl nets. In contrast, the second harvest showed the lowest FRAP values across all treatments, indicating that harvest time had a stronger effect on reducing power than shade treatment alone.

DPPH radical scavenging activity (EC50) ranged from 9.169 mg/mL (first harvest, red net) to 37.004 mg/mL (third harvest, blue net), with lower values indicating stronger antioxidant activity. The strongest activity was recorded in the first harvest under red nets, consistent with the high FRAP values under this treatment. In the second harvest, the blue-net treatment showed the best activity (EC50 = 13.391 mg/mL). In contrast, the third harvest exhibited the weakest DPPH activity across all treatments, especially under blue and red nets, despite their strong FRAP performance.

In our previous study, strong antioxidant activity was observed in all tested SEO samples from shaded sage leaves grown under blue nets. This research confirmed that sage responds positively to blue-light shading through increased production of secondary metabolic products such as EOs [11]. Cultivated sage was found to have stronger antioxidant activity (shaded plants, 6.16 mg/mL; non-shaded plants, 7.49 ± 0.13 mg/mL) compared to wild sage plants [12]. Similar antioxidant activity has been observed in SEO in studies from other countries. For example, SEO of plants from Tunisia showed an EC50 value of 6.7 mg/mL [33].

The relationship between the chemical composition of essential oil and observed antioxidant activity can be partly explained by the known bioactive properties of individual terpene constituents. Correlation analysis revealed that the EC_50_ was significantly negatively correlated with camphor (r = −0.62, *p* < 0.05), indicating that samples with higher camphor contents exhibited stronger DPPH radical scavenging activity. This is consistent with reports that camphor possesses moderate radical scavenging capacity due to its carbonyl group, which can participate in electron-transfer mechanisms. On the other hand, cis-thujone showed a significant positive correlation with EC50 (r = 0.61, *p* < 0.05), suggesting that higher thujone content is associated with weaker radical scavenging. This is in agreement with findings showing that thujone, as a saturated monoterpene ketone with limited hydrogen-donating capacity, contributes minimally to DPPH scavenging activity.

The divergent trends between FRAP and DPPH assays observed in the third harvest can be attributed to compositional differences in the sesquiterpene fraction. Viridiflorol and humulene epoxide II, which were strongly intercorrelated (r = 0.95, *p* < 0.01) and increased in the third harvest, are oxygenated sesquiterpenes with documented reducing potential. Viridiflorol, in particular, has been shown to exhibit moderate antioxidant activity in both DPPH and ABTS assays [34], while its electron-donating hydroxyl group enables participation in ferric ion reduction as measured by FRAP. This may explain the elevated FRAP values in third-harvest samples (particularly under blue and pearl nets) despite their weak DPPH performance, as the FRAP assay measures single-electron transfer capacity, whereas DPPH scavenging involves both hydrogen-atom and electron-transfer mechanisms.

1,8-Cineole, the third most abundant compound in sage EO, has well-documented antioxidant properties mediated through the Nrf2/Keap1 signaling pathway [35], yet its contribution to the overall antioxidant activity in the present study appeared limited, as no significant correlation was found between 1,8-cineole content and either FRAP or EC_50_ values. This suggests that the antioxidant activity of sage essential oil is determined by the synergistic or antagonistic interactions among multiple components rather than by any single dominant compound.

Multivariate analyses (PCA, HCA, and Pearson correlations) consistently identified distinct sample groupings based on essential oil composition and antioxidant activity. The first-harvest samples grown under pearl, red, and control nets, together with second-harvest samples grown under pearl and control nets, shared similar profiles characterized by higher camphor content (26.9–30.2%), moderate thujone content, and relatively high bornyl acetate levels. Their antioxidant activity was generally moderate, with EC50 values ranging from 16.9 to 30.3 mg/mL. In contrast, all third-harvest samples clustered together due to their elevated sesquiterpene contents (α-humulene, viridiflorol, and humulene epoxide II), lower camphor levels, and weaker DPPH scavenging activity (EC50: 28.9–37.0 mg/mL), likely driven by seasonal shifts in terpene biosynthesis toward the MVA pathway during late vegetative growth. The first-harvest blue-net sample grouped with the third-harvest cluster rather than with other first-harvest treatments, reflecting its distinctly low camphor (22.8%) and high thujone (39.5%) contents, which differentiated it compositionally from other first-harvest samples despite its superior essential oil yield. The second-harvest red- and blue-net samples formed a separate cluster, with H2 Blue exhibiting the most distinct overall profile due to its combination of the highest yield (3.29 mL/100 g), elevated trans-thujone content (13.1%), and strong antioxidant capacity (EC50 = 13.4 mg/mL). These groupings confirm that harvest time was the primary driver of compositional similarity, while blue-net shading consistently shifted samples away from the typical profile of their respective harvest period toward a more favorable thujone-to-camphor ratio.

Light, temperature, and plant density create a specific microenvironment that strongly affects sage biomass production, as well as essential oil yield and composition. Our results show that blue shading nets improved EO yield and quality, suggesting their potential use in protected cultivation of *Salvia officinalis*. Future studies should also evaluate sage cultivation under natural shading conditions, such as by intercropping with species of different growth habits, to develop practical recommendations for sustainable production. Sage essential oil has a wide range of applications as an antioxidant in pharmaceutical and cosmetic industries and as an antimicrobial agent in food processing. The highest yield of oil was obtained in the period of full flowering and the highest concentration of thujone.

The observed effects of shade nets on sage essential oil yield and composition can be interpreted through the lens of terpene biosynthesis regulation and light-mediated physiological responses. In Lamiaceae species, monoterpenes are synthesized predominantly via the plastidial methylerythritol phosphate (MEP) pathway, where 1-deoxy-D-xylulose 5-phosphate synthase (DXS) and 1-deoxy-D-xylulose 5-phosphate reductoisomerase (DXR) are rate-limiting enzymes whose expression is positively regulated by light [36,37]. Blue light (400–500 nm), perceived by cryptochromes and phototropins, has been shown to upregulate genes involved in the MEP pathway and to promote monoterpene accumulation in mint species, with blue-light supplementation increasing monoterpene content by up to 362% compared to controls without affecting glandular trichome density [38]. This is consistent with our observation that blue-net shading enhanced essential oil yield and favored thujone over camphor accumulation, particularly during the first and second harvests, when plants were in active flowering and trichome metabolic activity was at its peak.

Glandular trichomes are the primary sites of essential oil biosynthesis and storage in sage. Their density and metabolic activity are influenced by both developmental stage and environmental signals, with trichome formation being largely determined during early leaf expansion and metabolic output modulated throughout the organ’s lifespan [39]. The higher essential oil yields observed during the first harvest likely reflect the coincidence of peak flowering with maximal trichome secretory activity, while the progressive decline in yields under shading in the third harvest may have resulted from insufficient photosynthetically active radiation to sustain the carbon flux through the MEP pathway during a period of reduced day length and lower solar angle.

The shift from monoterpene-dominated to sesquiterpene-enriched profiles in the third harvest is consistent with a metabolic reallocation from the plastidial MEP pathway toward the cytosolic mevalonate (MVA) pathway, which supplies C15 precursor farnesyl diphosphate for sesquiterpene biosynthesis. Such shifts have been associated with reduced light availability and late-season senescence in aromatic plants [40]. The consistent reduction in camphor under blue nets across all harvests suggests a possible modulation of borneol dehydrogenase activity by blue-enriched light, although this hypothesis requires validation through gene expression studies.

## 5. Conclusions

The results of this study demonstrate that both shade-net color and harvest timing significantly influence essential oil yield, composition, and antioxidant activity in sage, with a strong interaction between the two factors. Blue shade nets proved most effective for enhancing essential oil yield and improving the thujone-to-camphor ratio during the first and second harvests, while shading became counterproductive in the third harvest, when reduced day length and light intensity favored open-field conditions. The initial flowering stage represents the optimal harvest time for maximizing both yield and essential oil quality. A three-harvest system with the last cut no later than the end of September supports sustained productivity in southern Serbian conditions. However, the economic feasibility of seasonal shade-net application was not evaluated and should be addressed in future research before definitive agronomic recommendations can be made.

## Figures and Tables

**Table 1:** Photosynthetically active radiation (PAR) and solar irradiance recorded under different shade-net treatments and in an open field (control) during the 2024 growing season in Moravac, southern Serbia.

Month	PAR (µmol m^−2^ s^−1^)				Solar Irradiance (W/m^2^)			
	Control	Pearl	Red	Blue	Control	Pearl	Red	Blue
May	1530.3	793.6	814.2	770.1	674.7	389.1	403.9	331.7
June	1928.0	978.6	1004.1	946.5	866.2	564.1	599.3	514.8
July	2025.8	1105.4	1193.9	1018.3	996.0	603.6	647.2	563.8
August	1867.4	961.7	993.6	937.2	789.1	483.8	501.1	442.0

Values represent monthly means recorded using a Watchdog 2475 data logger and Delta-T SunScan SS1 ceptometer. Measurements were taken at canopy level under each net and in the open field.

**Table 2:** Effect of colored shade nets on sage plant morphology and total yield.

Photoselective Nets	Plant Height (cm)	Leaf Width (cm)	Leaf Length (cm)	Fresh Mass per Plant (g)	Fresh Yield (t/ha^−1^)	Dry Yield (t/ha^−1^)	Fresh/Dry Yield Ratio
	First harvest of sage (at the end of May 2024)						
Pearl	54.1 ^b^	1.74 ^bc^	8.93 ^bc^	123.2 ^def^	5.790 ^def^	1.430 ^cd^	4.08
Red	58.2 ^ab^	1.78 ^ab^	9.51 ^ab^	136.1 ^d^	6.390 ^d^	1.602 ^c^	4.10
Blue	55.4 ^ab^	1.70 ^bc^	8.72 ^c^	130.7 ^de^	6.140 ^de^	1.517 ^cd^	4.03
Control	41.7 ^c^	1.66 ^c^	7.67 ^de^	107.1 ^efg^	5.030 ^efg^	1.249 ^de^	3.97
	Second harvest of sage (August 2024)						
Pearl	57.2 ^ab^	1.80 ^ab^	8.97 ^abc^	213.1 ^b^	10.012 ^b^	2.503 ^b^	4.16
Red	60.7 ^a^	1.89 ^a^	9.64 ^a^	256.5 ^a^	12.062 ^a^	3.114 ^a^	3.86
Blue	56.6 ^ab^	1.81 ^ab^	9.13 ^abc^	237.6 ^a^	11.208 ^a^	2.921 ^a^	3.81
Control	44.9 ^c^	1.63 ^c^	7.89 ^d^	179.3 ^c^	8.604 ^c^	2.267 ^b^	3.77
	Third harvest of sage (at the end of September)						
Pearl	26.3 ^d^	1.40 ^de^	7.6 ^ef^	104.2 ^fg^	4.897 ^ef^	1.208 ^de^	4.02
Red	25.7 ^de^	1.44 ^d^	7.52 ^ef^	113.0 ^def^	5.630 ^def^	1.290 ^cde^	4.10
Blue	24.8 ^de^	1.39 ^de^	7.18 ^f^	100.4 ^fg^	4.728 ^ef^	1.189 ^de^	3.96
Control	20.5 ^e^	1.30 ^e^	6.04 ^g^	84.6 ^g^	4.101 ^f^	1.048 ^e^	3.81
Harvest (H)	**	**	**	**	**	**	
Photoselective nets (PSN)	**	**	**	**	**	**	
H X PSN	NS	NS	NS	*	NS	NS	

Different lowercase letters within the column indicate significant differences among all harvest × net combinations according to Duncan’s multiple range test (*p* < 0.05). H—harvest time; NS—not significant. * significant at *p* < 0.05; ** significant at *p* < 0.01.

**Table 3:** Essential oil yield (mL/100 g plant material) of sage (*Salvia officinalis* L.) grown under different color shade nets across three harvest times.

Harvest/Nets	Essential Oil Yield, mL/100 g p.m.
**Harvest I**	
Pearl net	2.23 ± 0.103 ^e^
Red net	2.66 ± 0.052 ^d^
Blue net	4.09 ± 0.044 ^a^
Control	2.02 ± 0.064 ^f^
**Harvest II**	
Pearl net	2.13 ± 0.045 ^ef^
Red net	1.85 ± 0.051 ^g^
Blue net	3.29 ± 0.022 ^c^
Control	1.75 ± 0.045 ^gh^
**Harvest III**	
Pearl net	2.25 ± 0.095 ^e^
Red net	1.63 ± 0.044 ^h^
Blue net	1.87 ± 0.026 ^g^
Control	3.55 ± 0.162 ^b^
Harvest time (HT)	**
Shade net (SN)	**
HTXSN	**

Values are means ± standard deviation (*n* = 3). Different lowercase letters within a column indicate significant differences among all harvest × net combinations according to Duncan’s multiple range test (*p* < 0.05). HT—harvest time; SN—shade net treatment. ** indicates significance at *p* < 0.01.

**Table 4:** Essential oil composition of sage from first harvest.

N^o^	*t*_ret_, min	Compound	RI^exp^	RI^lit^	Method of Identification	Content %			
						Harvest I			
						Pearl	Red	Blue	Control
1.	6.27	Tricyclene	918	921	RI, MS	0.1	0.1	0.1	0.1
2.	6.37	α-Thujene	921	924	RI, MS	tr	tr	0.1	tr
3.	6.58	α-Pinene	929	932	RI, MS	3.4	2.8	3.5	2.3
4.	7.02	Camphene	944	946	RI, MS	-	4.1	2.8	3.5
5.	7.74	Sabinene	969	969	RI, MS	tr	tr	tr	tr
6.	7.85	β-Pinene	973	974	RI, MS, Co-I	1.3	1.1	1.2	1.1
7.	8.24	Myrcene	986	988	RI, MS	0.8	0.8	0.9	0.8
8.	9.14	α-Terpinene	1013	1014	RI, MS	0.1	0.1	0.2	0.1
9.	9.44	*o*-Cymene	1022	1022	RI, MS	0.3	0.3	0.3	0.3
10.	9.58	Limonene	1025	1024	RI, MS, Co-I	1.4	1.3	1.3	1.3
11.	9.69	1,8-Cineole	1028	1026	RI, MS, Co-I	11.0	10.0	9.6	9.0
12.	9.88	(*Z*)-β-Ocimene	1033	1032	RI, MS	-	-	tr	tr
13.	10.66	γ-Terpinene	1055	1054	RI, MS	0.2	0.2	0.2	0.2
14.	11.15	*cis*-Sabinene hydrate	1067	1065	RI, MS	-	tr	tr	tr
15.	11.79	Terpinolene	1085	1086	RI, MS	0.2	0.2	0.2	0.2
16.	12.63	*cis*-Thujone	1106	1101	RI, MS	30.5	27.3	31.6	27.3
17.	13.03	*trans*-Thujone	1116	1112	RI, MS	7.1	5.5	7.9	8.0
18.	14.00	iso-3-Thujanol	1139	1134	RI, MS	-	-	tr	tr
19.	14.22	Camphor	1145	1141	RI, MS, Co-I	26.9	29.9	22.8	29.1
20.	14.79	*trans*-Pinocamphone	1158	1158	RI, MS	0.2	0.2	0.2	0.2
21.	15.23	Borneol	1169	1165	RI, MS	3.8	3.3	2.7	3.5
22.	15.63	Terpinen-4-ol	1178	1174	RI, MS, Co-I	0.4	0.4	0.4	0.4
23.	16.24	α-Terpineol	1193	1186	RI, MS	0.2	0.3	0.2	0.2
24.	16.47	Myrtenol	1198	1194	RI, MS	-	0.1	0.2	tr
25.	19.98	Bornyl acetate	1280	1284	RI, MS	1.3	1.4	1.2	1.7
26.	20.30	*trans*-Sabinyl acetate	1288	1289	RI, MS	0.2	0.2	0.2	0.2
27.	25.54	(*E*)-Caryophyllene	1414	1417	RI, MS, Co-I	1.3	1.4	1.4	1.4
28.	26.33	Aromadendrene	1433	1439	RI, MS	-	tr	tr	tr
29.	26.93	α-Humulene	1448	1452	RI, MS	1.9	3.0	2.8	2.8
30.	32.02	Caryophyllene oxide	1578	1582	RI, MS	0.3	0.3	0.4	0.4
31.	32.46	Viridiflorol	1590	1592	RI, MS	1.7	3.4	3.6	3.6
32.	33.04	Humulene epoxide II	1605	1608	RI, MS	0.3	0.6	0.7	0.7
33.	47.97	Manool	2046	2056	RI, MS	0.7	1.9	1.9	1.9
	Total identified (%)					100.0			
	Grouped components (%)								
	Monoterpene hydrocarbons (1–9, 11, 12)					12.2	10.9	10.8	9.9
	Oxygen-containing monoterpenes (10, 13–21)					81.6	78.5	77.1	79.4
	Sesquiterpene hydrocarbons (22, 23)					3.2	4.3	6.0	4.2
	Oxygen-containing sesquiterpenes (24–26)					2.3	4.3	4.6	4.6
	Diterpenes (27)					0.7	1.9	1.5	1.9

*t*_ret._: retention time; RI^lit^: retention indices from the literature (Adams, 2007) [17]; RI^exp^: experimentally determined retention indices using a homologous series of *n*-alkanes (C_8_–C_20_ and C_21_–C_40_) on the HP-5MS column; MS: constituent identified by mass-spectral comparison; RI: constituent identified by retention index matching; Co-I: constituent identity confirmed by GC co-injection of an authentic sample; tr = trace amount (<0.05%).

**Table 5:** Essential oil composition of sage from second harvest.

N^o^	*t*_ret_, min	Compound	RI^exp^	RI^lit^	Method of Identification	Content, %			
						Harvest II			
						Pearl	Red	Blue	Control
1.	6.27	Tricyclene	918	921	RI, MS	0.1	0.1	0.1	0.1
2.	6.37	α-Thujene	921	924	RI, MS	0.1	0.1	0.1	0.1
3.	6.58	α-Pinene	929	932	RI, MS	3.3	2.5	2.9	2.6
4.	7.02	Camphene	944	946	RI, MS	4.5	3.8	3.8	4.2
5.	7.74	Sabinene	969	969	RI, MS	tr	tr	tr	0.1
6.	7.85	β-Pinene	973	974	RI, MS, Co-I	1.4	1.3	1.3	1.5
7.	8.01	1-Octen-3-ol	979	974	RI, MS	0.1	0.8	1.0	0.1
8.	8.24	Myrcene	986	988	RI, MS	0.9	0.1	0.1	0.8
9.	9.14	α-Terpinene	1013	1014	RI, MS	0.1	0.3	0.3	0.1
10.	9.44	*o*-Cymene	1022	1022	RI, MS	0.3	-	1.7	0.2
11.	9.58	Limonene	1025	1024	RI, MS, Co-I	1.5	1.3		1.4
12.	9.69	1,8-Cineole	1028	1026	RI, MS, Co-I	8.2	8.1	8.3	10.8
13.	9.88	(*Z*)-β-Ocimene	1033	1032	RI, MS	tr	0.1	-	0.2
14.	10.68	γ-Terpinene	1055	1054	RI, MS	0.2	0.2	0.2	0.1
15.	11.15	*cis*-Sabinene hydrate	1067	1065	RI, MS	0.1	**-**	0.1	0.2
16.	11.79	Terpinolene	1085	1086	RI, MS	0.2	0.2	0.2	0.2
17.	12.63	*cis*-Thujone	1106	1101	RI, MS	24.1	35.0	25.5	27.4
18.	13.03	*trans*-Thujone	1116	1112	RI, MS	10.7	8.5	13.1	4.9
19.	14.00	iso-3-Thujanol	1139	1134	RI, MS	0.1	0.1	0.1	tr
20.	14.22	Camphor	1145	1141	RI, MS, Co-I	29.0	25.5	24.7	30.2
21.	14.80	*trans*-Pinocamphone	1158	1158	RI, MS	0.2	tr	tr	tr
22.	15.23	Borneol	1169	1165	RI, MS	2.7	2.1	2.4	3.2
23.	15.63	Terpinen-4-ol	1178	1174	RI, MS, Co-I	0.4	0.4	0.4	0.5
24.	16.24	α-Terpineol	1193	1186	RI, MS	0.2	0.1	0.2	0.2
25.	16.47	Myrtenol	1198	1194	RI, MS	0.1	tr	tr	0.1
26.	19.11	iso-3-Thujanol acetate	1260	1267	RI, MS	-	tr	0.1	-
27.	19.98	Bornyl acetate	1280	1284	RI, MS	1.4	1.2	1.1	1.6
28.	20.30	*trans*-Sabinyl acetate	1288	1289	RI, MS	0.2	0.2	0.2	0.2
29.	25.54	(*E*)-Caryophyllene	1414	1417	RI, MS, Co-I	1.1	1.2	1.0	1.7
30.	26.33	Aromadendrene	1433	1439	RI, MS	tr	0.1	tr	0.2
31.	26.93	α-Humulene	1448	1452	RI, MS	2.6	1.9	3.0	2.8
32.	32.02	Caryophyllene oxide	1578	1582	RI, MS	0.3	0.3	0.3	0.4
33.	32.46	Viridiflorol	1590	1592	RI, MS	3.1	2.0	3.9	2.2
34.	33.04	Humulene epoxide II	1605	1608	RI, MS	0.7	0.4	0.9	0.4
35.	39.93	(*Z*)-α-*trans*-Bergamotol acetate	1797	1794	RI, MS	0.2	tr	0.3	tr
36.	47.97	Manool	2046	2056	RI, MS	1.9	2.0	2.7	1.3
	Grouped components (%)								
	Monoterpene hydrocarbons (1–6, 8–11, 13, 14, 16)					12.6	10.7	11.8	11.5
	Oxygen-containing monoterpenes (12, 15, 17–27)					77.4	81.3	76.0	79.4
	Sesquiterpene hydrocarbons (28–30)					3.7	3.2	4.0	4.7
	Oxygen-containing sesquiterpenes (31–34)					4.3	2.8	5.5	3.0
	Diterpenes (35)					1.9	2.0	2.7	1.3
	Others (7)					0.1	-	-	0.1

*t*_ret._: retention time; RI^lit^: retention indices from the literature [17]; RI^exp^: experimentally determined retention indices using a homologous series of *n*-alkanes (C_8_–C_20_) on the HP-5MS column; MS: constituent identified by mass-spectral comparison; RI: constituent identified by retention index matching; Co-I: constituent identity confirmed by GC co-injection of an authentic sample; tr = trace amount (<0.05%).

**Table 6:** Essential oil composition of sage from third harvest.

N^o^	*t*_ret_, min	Compound	RI^exp^	RI^lit^	Method of Identification	Content %			
						Harvest III			
						Pearl	Red	Blue	Control
1.	6.27	Tricyclene	918	921	RI, MS	0.1	0.1	0.1	0.1
2.	6.37	α-Thujene	921	924	RI, MS	0.1	0.1	0.1	0.1
3.	6.58	α-Pinene	929	932	RI, MS	4.4	2.5	3.5	2.9
4.	7.02	Camphene	944	946	RI, MS	4.2	3.9	3.1	3.9
5.	7.74	Sabinene	969	969	RI, MS	0.1	0.1	0.1	0.1
6.	7.85	β-Pinene	973	974	RI, MS, Co-I	1.9	1.7	1.8	2.0
7.	8.01	1-Octen-3-ol	979	974	RI, MS	0.1	tr	0.1	-
8.	8.24	Myrcene	986	988	RI, MS	1.0	0.7	0.8	0.7
9.	9.14	α-Terpinene	1013	1014	RI, MS	0.2	0.1	0.1	0.1
10.	9.44	*o*-Cymene	1022	1022	RI, MS	0.3	0.3	0.2	0.2
11.	9.58	Limonene	1025	1024	RI, MS, Co-I	1.4	1.1	1.1	1.0
12.	9.69	1,8-Cineole	1028	1026	RI, MS, Co-I	8.0	9.5	10.6	9.9
13.	9.86	(*Z*)-β-Ocimene	1033	1032	RI, MS	0.2	-	tr	tr
14.	10.68	γ-Terpinene	1055	1054	RI, MS	0.3	0.2	0.3	0.2
15.	11.15	*cis*-Sabinene hydrate	1067	1065	RI, MS	0.1	0.1	0.1	0.1
16.	11.79	Terpinolene	1085	1086	RI, MS	0.2	0.1	0.2	0.2
17.	12.63	*cis*-Thujone	1106	1101	RI, MS	32.6	36.1	34.3	28.0
18.	13.03	*trans*-Thujone	1116	1112	RI, MS	8.0	6.6	8.3	9.6
19.	14.00	iso-3-Thujanol	1139	1134	RI, MS	tr	tr	0.1	tr
20.	14.22	Camphor	1145	1141	RI, MS, Co-I	23.4	22.5	20.0	22.2
21.	14.80	*trans*-Pinocamphone	1158	1158	RI, MS	0.2	0.2	0.2	0.2
22.	15.23	Borneol	1169	1165	RI, MS	2.9	2.7	2.4	2.7
23.	15.63	Terpinen-4-ol	1178	1174	RI, MS, Co-I	0.4	0.4	0.5	0.4
24.	16.24	α-Terpineol	1193	1186	RI, MS	0.2	tr	0.2	0.2
25.	16.47	Myrtenol	1198	1194	RI, MS	0.2	tr	0.1	0.1
26.	19.98	Bornyl acetate	1280	1284	RI, MS	0.8	0.6	0.9	0.7
27.	20.30	*trans*-Sabinyl acetate	1288	1289	RI, MS	0.2	0.2	0.2	0.1
28.	25.54	(*E*)-Caryophyllene	1414	1417	RI, MS, Co-I	1.6	1.2	1.0	2.7
29.	26.33	Aromadendrene	1433	1439	RI, MS	0.1	-	tr	0.2
30.	26.93	α-Humulene	1448	1452	RI, MS	2.5	2.2	2.5	4.7
31.	32.02	Caryophyllene oxide	1578	1582	RI, MS	0.3	0.4	0.4	0.3
32.	32.46	Viridiflorol	1590	1592	RI, MS	2.7	3.5	3.5	3.8
33.	33.04	Humulene epoxide II	1605	1608	RI, MS	0.5	0.7	0.7	0.7
34.	47.97	Manool	2046	2056	RI, MS	1.0	2.3	2.3	1.9
						100.0	100.0	100.0	100.0
	Monoterpene hydrocarbons (1–6, 8–11, 13, 14, 16)					14.3	10.8	11.4	11.5
	Oxygen-containing monoterpenes (12, 15, 17–27)					76.9	78.9	78.1	74.2
	Sesquiterpene hydrocarbons (28–30)					4.2	3.4	3.5	7.6
	Oxygen-containing sesquiterpenes (31–33)					3.5	4.6	4.5	4.8
	Diterpenes (34)					1.0	2.3	2.3	1.9
	Others (7)					0.1	tr	0.1	-

*t*_ret._: retention time; RI^lit^: retention indices from the literature [17]; RI^exp^: experimentally determined retention indices using a homologous series of *n*-alkanes (C_8_–C_20_) on the HP-5MS column; MS: constituent identified by mass-spectral comparison; RI: constituent identified by retention index matching; Co-I: constituent identity confirmed by GC co-injection of an authentic sample; tr = trace amount (<0.05%).

**Table 7:** The contents of seven major essential oil compounds.

Photoselective Net	*cis*- Thujone	*trans*- Thujone	Camphor	1,8- Cineole	Borneol	α-Humulene	Viridiflorol
First harvest of sage (at the end of May 2024)							
Pearl	30.4 ^f^	7.04 ^g^	26.8 ^c^	10.7 ^a^	3.75 ^a^	1.89 ^h^	1.68 ^i^
Red	27.7 ^g^	5.46 ^i^	29.6 ^ab^	10.0 ^b^	3.31 ^c^	3.03 ^c^	3.43 ^d^
Blue	32.0 ^d^	7.97 ^f^	23.1 ^e^	9.6 ^c^	2.67 ^f^	3.79 ^b^	3.57 ^c^
Control	27.4 ^g^	8.08 ^ef^	29.0 ^b^	8.9 ^e^	3.55 ^b^	2.77 ^e^	3.52 ^cd^
Second harvest of sage (August 2024)							
Pearl	24.0 ^i^	10.49 ^b^	29.1 ^b^	8.2 ^f^	2.72 ^f^	2.58 ^f^	3.10 ^e^
Red	35.1 ^b^	8.54 ^d^	25.4 ^d^	8.2 ^f^	2.08 ^i^	1.93 ^h^	2.01 ^h^
Blue	25.6 ^h^	13.13 ^a^	24.8 ^d^	8.3 ^f^	2.40 ^g^	2.93 ^d^	3.89 ^a^
Control	27.4 ^g^	4.89 ^j^	30.2 ^a^	10.8 ^a^	3.17 ^d^	2.81 ^e^	2.22 ^g^
Third harvest of sage (at the end of September)							
Pearl	32.3 ^d^	8.03 ^ef^	23.2 ^e^	8.1 ^f^	2.86 ^e^	2.53 ^f^	2.69 ^f^
Red	36.4 ^a^	6.64 ^h^	22.2 ^f^	9.4 ^c^	2.72 ^f^	2.14 ^g^	3.56 ^c^
Blue	34.1 ^c^	8.33 ^de^	20.3 ^g^	10.6 ^a^	2.37 ^h^	2.53 ^f^	3.50 ^cd^
Control	27.5 ^g^	9.64 ^c^	22.0 ^f^	10.0 ^b^	2.67 ^f^	4.74 ^a^	3.81 ^b^
Harvest (H)	**	**	**	**	**	**	**
Photoselective nets (SN)	**	**	**	**	**	**	**
H X SN	**	**	**	**	**	**	**

Values are means ± standard deviation (*n* = 3). Different lowercase letters within a column indicate significant differences among all harvest × net combinations according to Duncan’s multiple range test (*p* < 0.05). H—harvest time; SN—shade net treatment. ** indicates significance at *p* < 0.01.

**Table 8:** Antioxidant activity of sage (*Salvia officinalis* L.) essential oils under different colors of shade net treatments across three harvest times, as determined by FRAP and DPPH assays.

Sample/Net	FRAP Value, mg Fe^2+^/g Oil	EC50, mg/mL
Harvest I		
Pearl net	0.519 ± 0.050 ^g^	30.339 ± 0.155 ^c^
Red net	1.151 ± 0.005 ^a^	9.169 ± 0.039 ^k^
Blue net	0.653 ± 0.002 ^e^	19.129 ± 0.105 ^h^
Control	0.486 ± 0.002 ^hi^	16.857 ± 0.046 ^i^
Harvest II		
Pearl net	0.466 ± 0.002 ^i^	24.223 ± 0.096 ^e^
Red net	0.462 ± 0.002 ^i^	22.655 ± 0.062 ^g^
Blue net	0.563 ± 0.002 ^f^	13.391 ± 0.045 ^j^
Control	0.507 ± 0.002 ^gh^	23.071 ± 0.101 ^f^
Harvest III		
Pearl net	0.951 ± 0.002 ^c^	28.880 ± 0.090 ^d^
Red net	0.721 ± 0.002 ^d^	36.677 ± 0.242 ^b^
Blue net	1.123 ± 0.005 ^b^	37.004 ± 0.251 ^a^
Control	0.559 ± 0.003 ^f^	30.475 ± 0.113 ^c^
Harvest time (HT)	**	**
Shade nets (SN)	**	**
HTXSN	**	**

Values are means ± standard deviation (*n* = 3). FRAP values are expressed as mg Fe^2+^ equivalents per g of essential oil; EC50 values represent the concentration (mg/mL) required to scavenge 50% of DPPH radicals after 20 min, with lower values indicating higher activity. Different lowercase letters within each column indicate significant differences among all harvest × net combinations according to Duncan’s multiple range test (*p* < 0.05). HT—harvest time; SN—shade-net treatment; ** indicates significance at *p* < 0.01.

**Table 9:** Pearson’s correlation coefficients among essential oil yield, antioxidant activity parameters, and major essential oil components of sage (*Salvia officinalis* L.).

	EO Yield	FRAP	EC50	*cis*-Thujone	*trans*-Thujone	Camphor	1,8-Cineole	α-Humulene	Viridiflorol
EO yield	1								
FRAP	−0.04	1							
EC_50_	−0.38	0.08	1						
*cis*-Thujone	−0.30	0.31	0.61 *	1					
*trans*-Thujone	0.39	−0.31	−0.11	−0.34	1				
Camphor	−0.27	−0.31	−0.62 *	−0.64 *	−0.28	1			
1,8-Cineole	−0.04	0.20	0.27	0.08	−0.59 *	0.01	1		
α-Humulene	0.60 *	−0.01	−0.14	−0.47	0.24	−0.12	0.10	1	
Viridiflorol	0.49	0.27	−0.17	−0.22	0.41	−0.35	−0.16	0.59 *	1
Humulene epoxide II	0.44	0.12	−0.19	−0.28	0.62 *	−0.32	−0.31	0.45	0.95 **

* Significant at *p* < 0.05; ** significant at *p* < 0.01.

## Data Availability

All the data are available in the manuscript file.

## Supplementary Materials

The following supporting information can be downloaded at: https://www.mdpi.com/article/10.3390/plants15111711/s1, Table S1: Two-way ANOVA results for major essential oil compounds of sage (*Salvia officinalis* L.) as affected by harvest time (H), shade net treatment (SN), and their interaction (H × SN); Table S2: Two-way ANOVA results for morphological parameters of sage (*Salvia officinalis* L.) as affected by harvest time (H), shade net treatment (SN), and their interaction (H × SN).
