# Supplementary material for: Influence of Shading on Essential Oil Quantity and Quality of Sage (Salvia officinalis L.) at Different Harvest Times
Source: Plants (Basel). 2026 Jun 1;15(11):1711. doi: 10.3390/plants15111711 (PMC13259298; doi:10.3390/plants15111711)
Supplement: Supplementary file 1 [file plants-15-01711-s001.zip › plants-4326679-supplementary.pdf]

Supplementary Table S1. Two-way ANOVA results for major essential oil compounds of sage (*Salvia officinalis* L.) as affected by harvest time (H), shade net treatment (SN), and their interaction (H × SN).

**Source**	**df**	***cis*-Thujone**			***trans*-Thujone**			**Camphor**			**1,8-Cineole**			**Borneol**			**α-Humulene**			**Viridiflorol**		
		MS	F	p	MS	F	p	MS	F	p	MS	F	p	MS	F	p	MS	F	p	MS	F	p
Harvest (H)	2	65.12	266.4	<0.001	13.564	454.29	<0.001	113.51	500.5	<0.001	2.491	68.43	<0.001	1.9554	690.8	<0.001	0.5731	217.9	<0.001	1.0388	238.50	<0.001
Shade nets (SN)	3	52.77	215.9	<0.001	14.670	491.32	<0.001	32.66	144.0	<0.001	1.375	37.76	<0.001	0.9094	321.3	<0.001	2.6907	1022.9	<0.001	2.0879	479.37	<0.001
H × SN	6	38.13	156.0	<0.001	15.241	510.43	<0.001	9.50	41.9	<0.001	4.324	118.77	<0.001	0.2662	94.0	<0.001	1.9879	755.7	<0.001	1.6872	387.37	<0.001
Error	24	0.24			0.030			0.23			0.036			0.0028			0.0026			0.0044		

Supplementary Table S2. Two-way ANOVA results for morphological parameters of sage (*Salvia officinalis* L.) as affected by harvest time (H), shade net treatment (SN), and their interaction (H × SN).

**Source**	**df**	**Plant height (cm)**			**Leaf width (cm)**			**Leaf height (cm)**			**Fresh mass per plant (g)**		
		MS	F	p	MS	F	p	MS	F	p	MS	F	p
Harvest (H)	2	3446.85	379.99	<0.001	0.5556	142.16	<0.001	11.988	84.13	<0.001	49361.7	286.66	<0.001
Shade nets (SN)	3	277.42	30.58	<0.001	0.0470	12.02	<0.001	4.747	33.31	<0.001	3249.0	18.87	<0.001
H × SN	6	22.52	2.48	0.052	0.0037	0.94	0.482	0.102	0.72	0.640	500.3	2.91	0.028
Error	24	9.07			0.0039			0.142			172.2		
